# Impact of general anesthesia on postoperative complications in orthognathic surgery: a retrospective comparison of total intravenous anesthesia versus volatile anesthesia

**DOI:** 10.1038/s41598-024-66926-w

**Published:** 2024-07-12

**Authors:** Reona Aijima, Daisuke Miura, Ayako Takamori, Asana Kamohara, Atsushi Danjo, Yoshiro Sakaguchi, Yoshio Yamashita

**Affiliations:** 1 Department of Oral and Maxillofacial Surgery, Faculty of Medicine, Saga University, 5-1-1 Nabeshima, Saga, 849-8501 Japan; 2 Department of Anesthesiology, Saga Medical Center Koseikan, 400, Nakabaru, Kasemachi, Saga, Japan; 3 Clinical Research Center, Saga University Hospital, 5-1-1 Nabeshima, Saga, Japan; 4 Department of Anesthesiology and Critical Care Medicine, Faculty of Medicine, Saga University, 5-1-1 Nabeshima, Saga, Japan

**Keywords:** Medical research, Risk factors

## Abstract

Orthognathic surgery has a high incidence of postoperative nausea (PON) and vomiting (POV), delaying mobility initiation and postoperative recovery. Bleeding is another risk associated with this surgical procedure. We aimed to compare total intravenous anesthesia (TIVA) and volatile anesthesia in patients undergoing orthognathic surgery in terms of postoperative nausea and vomiting (PONV) incidence and hemodynamic changes. This retrospective study included 82 patients who underwent bilateral sagittal split ramus osteotomies at Saga University Hospital between April 2016 and April 2021. We compared the effects of TIVA and volatile anesthesia on PONV onset after surgery, acute postoperative hemodynamic changes (blood pressure and heart rate), and factors contributing to PONV. PON was significantly lower in the TIVA group than in the volatile anesthesia group. The total dose of fentanyl contributed to the onset of POV, while the onset of PON was associated with low volumes of fluid infusion and urine in the TIVA and volatile anesthesia groups, respectively. Furthermore, post-extubation hemodynamic change was significantly smaller in the TIVA group than in the volatile anesthesia group. Therefore, TIVA could have a reduced risk of PONV and hemodynamic changes in patients undergoing orthognathic surgery. Employing TIVA could mitigate perioperative complications and enhance patient safety.

## Introduction

Orthognathic surgery is often performed in young individuals. Intra- and postoperative management are important and greatly affect the patient’s quality of life during hospital stays. Postoperative nausea and vomiting (PONV) is one of the most unpleasant experiences after general anesthesia and is considered more intolerable than wound pain^1,2^. PONV is the most common anesthesia-related complication, and its persistence has been linked to serious complications, such as suture dehiscence, postoperative bleeding, esophageal rupture, subcutaneous emphysema, pneumothorax, and increased intracranial pressure^3,4^. In addition, the occurrence of PONV is associated with a significantly longer stay in the post-anesthesia care unit, unanticipated hospital admissions, and increased healthcare costs^2^. The incidence of PONV after orthognathic surgery is higher than that after other stomatognathic surgical procedures, such as fractures and tooth extractions, and this is also important from the viewpoint of securing the airway in the event of sudden changes in the patient’s condition^5^. Risk factors for the incidence of PONV are classified into three categories: patient-specific, anesthetic, and surgical factors^2–4^.

Regarding orthognathic surgery, volatile anesthesia (where agents, such as sevoflurane, are administered) or total intravenous anesthesia (TIVA), where anesthesia is initiated and maintained using an intravenous anesthetic (propofol), is commonly employed. TIVA has been reported to reduce the risk of PONV compared with volatile anesthesia^2,6^. Although there is a report on risk factors for PONV in patients undergoing orthognathic surgery, the effectiveness of TIVA has not been investigated^7^. In these patients, sex, smoking status, predisposing conditions, and opioid use have been shown to influence the occurrence of PONV^8^, consistent with the findings reported by Apfel et al.^3^. In addition, although there is a report describing the influence of age, surgical site, operation time, and type of anesthesia, the relationship between patient background and risk factors remains unclear^8^. This study aimed to investigate the effect of general anesthesia on the incidence of PONV in patients undergoing orthognathic surgery and its relationship with other relevant factors by performing a multivariate analysis.

Emergence and extubation significantly increase blood pressure (BP) and heart rate (HR), resulting in perioperative complications, such as postoperative bleeding^9^. Emergence agitation (EA) is less likely to occur after TIVA than after volatile anesthesia^10,11^. Although TIVA is expected to reduce the changes in hemodynamics during emergence and extubation, the effects of TIVA and inhaled anesthetics on circulatory dynamics have not been reported. Therefore, we also verified the effect of general anesthesia on the hemodynamic response during emergence.

## Methods

### Study design and ethical approval

This study was approved by the Institutional Review Board of Saga University Hospital (approval number: 2022-04-R-02) and conducted in accordance with the Declaration of Helsinki. The need to obtain informed consent from the study participants was waived by the Institutional Review Board of Saga University Hospital due to the retrospective nature of the study. Patients who were eligible for this study had the opportunity to refuse to participate by opting out. Given the situation in our hospital, 51 patients with TIVA and 31 patients with volatile anesthesia, totaling 82 patients, were recruited for this study. Based on previous studies^7,8,12,13^ and the clinical situation at our hospital over several months, the incidence of postoperative nausea (PON) was assumed to be approximately 35% and 65% for the TIVA and volatile anesthesia groups, respectively. Then, the power was calculated to be approximately 80% under a 5% significance level. Although postoperative vomiting (POV) was expected to be very rare in our hospital, it was considered important to include in this study. Therefore, the strategy was to enroll as many POV cases as possible. The inclusion criteria were patients who underwent bilateral sagittal split ramus osteotomies at Saga University Hospital between April 2016 and April 2021. The exclusion criteria were: (1) American Society of Anesthesiology physical status III or VI, (2) Patients receiving opioids via continuous infusion postoperatively, and (3) Patients who declined to participate in the study. One oral surgeon retrospectively investigated anesthesia records and hospitalization charts of the patients. PONV was defined as nausea and vomiting occurring within 48 h postoperatively and retrospectively determined from reviewing medical records. The primary endpoints were the presence or absence of PONV up to 48 h postoperatively and changes in hemodynamics pre- and post-extubation. With regard to hemodynamics, the pre- and intraoperative mean BP (systolic and diastolic BP), mean HR, and maximum values of the BP and HR postoperatively were investigated. The rate of change in the BP and HR was also examined during and after emergence [(highest postoperative value − intraoperative mean)/intraoperative mean × 100].

### General anesthesia and perioperative management

Rapid induction with propofol was performed in all 82 cases; after tracheal intubation, anesthesia was maintained with intravenous propofol in 51 cases (4–6 mg/kg/h) and inhaled anesthetics in 31. In the volatile anesthesia group, sevoflurane was used in 29 cases (1.0–2.0 vol%) and desflurane in two (5.0–6.0 vol%) (Supplementary Table S1). In the TIVA group, the bispectral index was used to monitor awareness with a target value of 40–60 (QE-910P, Nihon Kohden Corporation, Tokyo, Japan). Eight patients in the TIVA group received midazolam as needed. In both groups, fentanyl and rocuronium were administered ad libitum in addition to continuous remifentanil administration during anesthesia maintenance. The patients’ characteristics and risk factors are presented in Table 1. The average age of the participants was 25.6 years, comprising 30 males and 52 females.

**Table 1:** Patient characteristics and risk factors in the anesthesia groups (TIVA or volatile anesthesia).

Anesthesia	TIVA (n = 51)	Volatile anesthetics (n = 31)	OR (95% CI)	*P value*
Sex (male/female)	15/36	15/16	Reference (male) 2.25 (0.89–5.68)	0.086
Age (years)	23.6 ± 7.4	29.0 ± 11.6	0.94 (0.89–0.99)	0.018*
Smoking (Yes/No)	4/47	5/26	Reference (No) 0.44 (0.11–1.79)	0.254
BMI (kg/m^2^)	21.3 ± 2.4	21.4 ± 2.7	0.97 (0.81–1.16)	0.759
Surgical time (min)	175.6 ± 35.8	173.4 ± 33.5	1.00 (0.99–1.01)	0.782
Anesthesia time (min)	300.2 ± 42.4	297.3 ± 36.1	1.00 (0.99–1.01)	0.748
Use of opioids				
Fentanyl (μg)	284.8 ± 90.1	301.6 ± 80.6	0.89 (0.69–1.16)	0.397
Remifentanil (μg)	4923.0 ± 1341.6	4444.4 ± 1416.6	1.03 (0.99–1.07)	0.131
In–Out balance (mL)	1186.8 ± 520.1	1625.7 ± 608.7	0.87 (0.79–0.95)	0.003*
Infusion (mL)	2500.6 ± 786.8	2530.7 ± 619.7	0.99 (0.93–1.06)	0.854
Out (mL)	1313.8 ± 682.0	905.0 ± 409.6	1.18 (1.05–1.32)	0.005*
Bleeding (mL)	246.1 ± 133.5	273.8 ± 204.3	0.99 (0.96–1.02)	0.456
Urine (mL)	1067.7 ± 687.6	631.1 ± 366.9	1.21 (1.07–1.37)	0.002*

Mean ± S.D. *P value < 0.05.

Odds ratios (ORs) are based on the following one unit change: fentanyl 50 μg, remifentanil 100 μg, in–out balance (infusion and out) 100 mL, urine 100 mL, and bleeding 10 mL.

BMI, body mass index; CI, confidence interval; S.D., standard deviation; TIVA, total intravenous anesthesia.

Osteotomies were performed on both sides by three oral surgeons, according to the Obwegeser–Dal Pont method. All patients were administered 6.6 mg of dexamethasone on the day of surgery and the day after surgery to prevent PONV and postoperative local swelling. All patients underwent nasotracheal tube feeding due to intermaxillary fixation. Therefore, an 8-Fr gastric tube was inserted through the nasal route before extubation, and a chest X-ray confirmed that the tip was in the stomach, not the trachea.

### Statistical analyses

To clarify differences in patient characteristics and potential risk factors between the two groups (TIVA vs. volatile anesthesia), Fisher’s exact test or t-test was performed. Thereafter, to investigate potential risk factors for PONV, uni- and multivariate analyses were performed using logistic regression models. The objective variable was considered to be PON (+ or −), with explanatory variables including the type of anesthesia (TIVA or volatile), sex, age, smoking status, body mass index (BMI), surgery time, anesthesia time, total opioid dose (fentanyl and remifentanil), fluid infusion volume, out volume (bleeding volume + urine volume), bleeding volume, and urine volume. If the Pearson correlation coefficient was ≥ 0.6, the representative explanatory variable was included in multivariate analyses. Considering POV (+ or −), uni- and multivariate analyses were also performed using the logistic regression model, as described previously. Furthermore, to determine the optimal fentanyl cutoff value for POV risk (+ or −) and to calculate the area under the curve (AUC), a receiver operating characteristic (ROC) curve was generated. Additionally, logistic regression models were applied to PONV (PON and POV) within each group stratified by anesthesia method. Moreover, logistic regression analyses were performed to assess the effect of anesthesia on hemodynamics for the two groups, considering each factor. The *P*-values were all two-sided, with < 0.05 indicating statistical significance. All statistical analyses were performed using JMP Pro Version 15 (SAS Institute Inc., Cary, NC, USA).

### Ethics approval and consent to participate

This study was approved by the Institutional Review Board of Saga University School of Medicine (approval number 2022-04-R-02).

## Results

There were no differences in sex ratio, smoking, BMI, surgery and anesthesia times, opioid use, or infusion volume between the TIVA and volatile anesthesia groups. However, patients in the TIVA group were younger and had more urine output than those in the volatile anesthesia group (Table 1).

**Figure 1:**
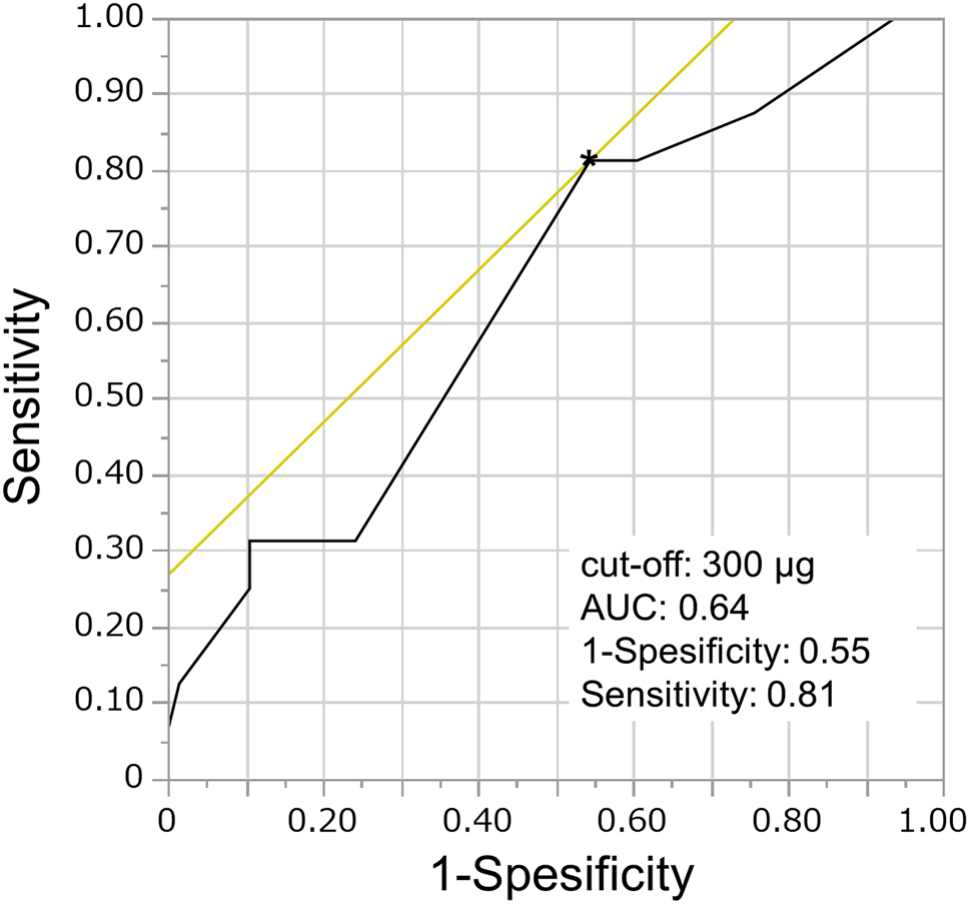
Receiver operating characteristic (ROC) curve of the association between postoperative vomiting (POV) and fentanyl. The cutoff value is 300 μg.

The effect of anesthesia on PON and POV was assessed. PON occurred in 67.7% of the patients in the volatile anesthesia group. However, PON occurrence was significantly lower in the TIVA group at 31.4% (odds ratio [OR]: 4.59, 95% confidence interval [CI] 1.76–11.97; *P* = 0.002) (Table 2). Additionally, it was clarified that low urine output was associated with the onset of PON (*P* = 0.023). There was no difference in POV between the two groups; the total dose of fentanyl was involved in the onset of POV (*P* = 0.039) (Table 3). Furthermore, the optimal fentanyl cutoff value of 300 μg for POV risk and an AUC of 0.6 were estimated using the maximum Youden index^14^ based on the ROC (Fig. 1).

**Table 2:** Relationships between PON risk with the anesthesia groups (TIVA or volatile anesthesia) and other factors using uni- and multivariate logistic regression analyses.

	PON +	PON-	Univariate		Multivariate	
	(n = 37)	(n = 45)	OR (95% CI)	*P value*	OR (95% CI)	*P value*
Anesthesia				0.002*		0.011*
TIVA	16 (43.2%)	35 (77.8%)	Reference		Reference	
Volatile anesthetics	21 (56.8%)	10 (22.2%)	4.59 (1.76–11.97)		5.33 (1.47–19.36)	
Sex				0.831		0.492
Male	14 (37.8%)	16 (35.6%)	Reference		Reference	
Female	23 (62.2%)	29 (64.4%)	0.91 (0.37–2.23)		1.58 (0.43–5.87)	
Smoking				0.456		0.343
Yes	3 (8.1%)	6 (13.3%)	Reference		Reference	
No	34 (91.9%)	39 (86.7%)	1.74 (0.40–7.51)		2.31 (0.41–12.94)	
Age	25.5	25.8	1.00 (0.95–1.04)	0.899	0.99 (0.93–1.06)	0.793
BMI (kg/m^2^)	21.3	21.4	0.98 (0.82–1.17)	0.853	1.04 (0.80–1.34)	0.778
Surgical time (min)	176.3	173.5	1.00 (0.99–1.02)	0.712		−
Anesthesia time (min)	298.5	299.6	1.00 (0.99–1.01)	0.904	1.00 (0.98–1.02)	0.890
Use of opioids						
Fentanyl (μg)	306.1	278.9	1.21 (0.92–1.60)	0.168	1.22 (0.88–1.71)	0.239
Remifentanil (μg)	4539.6	4908.6	0.98 (0.95–1.01)	0.230	0.99 (0.93–1.05)	0.786
In–Out balance	1367.2	1340.8	1.01 (0.93–1.09)	0.840		−
Infusion (mL)	2363.7	2633.9	0.95 (0.89–1.01)	0.098	0.94 (0.85–1.04)	0.227
Out (mL)	996.5	1293	0.91 (0.83–1.00)	0.041*		−
Bleeding (mL)	273.9	242.4	1.01 (0.99–1.04)	0.384	1.01 (0.97–1.05)	0.745
Urine (mL)	722.6	1050.7	0.89 (0.81–0.98)	0.023*	0.97 (0.83–1.12)	0.655

Odds ratios (ORs) are based on the following one unit change: fentanyl 50 μg, remifentanil 100 μg, in–out balance (infusion and out) 100 mL, urine 100 mL, and bleeding 10 mL.

*BMI* Body mass index, *CI* Confidence interval, *PON* Postoperative nausea, *TIVA* Total intravenous anesthesia.

*P *value* < 0.05.

**Table 3:** Relationships between POV risk with the anesthesia groups (TIVA or volatile anesthesia) and other factors using uni- and multivariate logistic regression analyses.

	POV +	POV −	Univariate		Multivariate	
	(n = 16)	(n = 66)	OR (95% CI)	*P value*	OR (95% CI)	*P value*
Anesthesia				0.585		0.741
TIVA	9 (56.25%)	42 (63.6%)	Reference		Reference	
Volatile anesthetics	7 (43.75%)	24 (36.4%)	1.36 (0.45–4.12)		0.78 (0.17–3.52)	
Sex				0.933		0.395
Male	6 (37.5%)	24 (36.4%)	Reference		Reference	
Female	10 (62.5%)	42 (63.6%)	0.95 (0.31–2.95)		2.06 (0.39–10.89)	
Smoking				0.828		0.82
Yes	2 (12.5%)	7 (10.6%)	Reference		Reference	
No	14 (87.5%)	59 (89.4%)	0.83 (0.16–4.44)		0.79 (0.10–5.97)	
Age	26.3	25.5	1.01 (0.95–1.07)	0.751	1.06 (0.97–1.15)	0.222
BMI (kg/m^2^)	21.7	21.2	1.08 (0.87–1.34)	0.495	1.11 (0.82–1.52)	0.496
Surgical time (min)	188.4	171.4	1.01 (1.00–1.03)	0.085		−
Anesthesia time (min)	306.6	297.3	1.01 (0.99–1.02)	0.400	1.01 (0.99–1.03)	0.383
Use of opioids						
Fentanyl (μg)	335.9	280.3	1.44 (1.02–2.05)	0.039*	1.71 (1.10–2.65)	0.017*
Remifentanil (μg)	4733.9	4744.1	1.00 (0.96–1.04)	0.979	0.98 (0.92–1.06)	0.652
In–out balance	1318.4	1361.1	0.99 (0.90–1.09)	0.795		–
Infusion (mL)	2417.5	2534.9	0.98 (0.90–1.06)	0.559	0.96 (0.85–1.08)	0.501
Out (mL)	1099.1	1173.8	0.98 (0.89–1.08)	0.666		–
Bleeding (mL)	300.9	245.8	1.02 (0.99–1.05)	0.232	1.01 (0.97–1.06)	0.518
Urine (mL)	798.1	928	0.96 (0.87–1.07)	0.454	0.98 (0.82–1.18)	0.831

Odds ratios (ORs) are based on the following one unit change: fentanyl 50 μg, remifentanil 100 μg, in–out balance (infusion and out) 100 mL, urine 100 mL, and bleeding 10 mL.

*BMI* Body mass index, *CI* Confidence interval, *POV* Postoperative vomiting, *TIVA* Total intravenous anesthesia.

*P value < 0.05.

The correlation between background factors was evaluated, and correlations were found between urine volume and output volume (correlation coefficient, r > 0.9), infusion volume and urine volume (r = 0.6), output volume (r = 0.6), and in–out balance (r = 0.6), and anesthesia duration and surgery duration (r > 0.9). Therefore, the multivariate analysis excluded output volume, in–out balance, and surgery time. PON was significantly affected by anesthesia (OR: 5.33, 95% CI 1.47–19.36; *P* = 0.011), and POV was significantly affected by the total dose of fentanyl (OR: 1.71, 95% CI 1.10–2.65; *P* = 0.017).

A stratified analysis of each anesthesia method revealed that low fluid volume in the TIVA group (*P* = 0.035) and low urine volume in the volatile anesthesia group (*P* = 0.017) were involved in PON (Table 4). Although there were no obvious risk factors for POV in the stratified analysis of the anesthesia method, high BMI was associated with POV in the inhalation anesthesia group (*P* = 0.056).

**Table 4:** PONV risk stratified by the anesthesia groups (TIVA or volatile anesthesia) based on logistic regression models.

	TIVA				Volatile anesthetics			
	PON		POV		PON		POV	
	OR (95%CI)	*P value*	OR (95% CI)	*P value*	OR (95% CI)	*P value*	OR (95% CI)	*P value*
Age (years)	1.00 (0.89–1.11)	0.954	1.05 (0.92–1.20)	0.450	0.97 (0.85–1.09)	0.593	1.01 (0.88–1.17)	0.841
BMI (kg/m^2^)	0.99 (0.69–1.41)	0.936	0.87 (0.55–1.36)	0.539	1.05 (0.66-–1.66)	0.838	3.01 (0.98–9.26)	0.056
Anesthesia Time (min)	1.00 (0.98–1.02)	0.795	1.01 (0.98–1.03)	0.659	1.03 (0.98–1.07)	0.208	1.03 (0.97–1.09)	0.342
Use of opioids								
Fentanyl (μg)	1.34 (0.88–2.03)	0.169	1.46 (0.93–2.30)	0.104	0.79 (0.31–1.97)	0.607	3.40 (0.51–22.53)	0.205
Remifentanil (μg)	1.00 (0.93–1.07)	0.904	0.99 (0.91–1.08)	0.801	1.00 (0.90–1.11)	0.979	0.89 (0.74–1.07)	0.203
Infusion (mL)	0.82 (0.69–0.99)	0.035*	0.90 (0.75–1.08)	0.255	1.05 (0.86-–1.28)	0.631	1.00 (0.80–1.25)	0.997
Bleeding (mL)	1.04 (0.98–1.11)	0.198	1.03 (0.96–1.11)	0.378	0.97 (0.91–1.05)	0.488	0.98 (0.91–1.06)	0.676
Urine (mL)	1.16 (0.96–1.40)	0.115	1.10 (0.90–1.35)	0.353	0.56 (0.35–0.90)	0.017*	0.46 (0.19–1.13)	0.092

Odds ratios (ORs) are based on the following one unit change: fentanyl 50 μg, remifentanil 100 μg, infusion 100 mL, urine 100 mL, and bleeding 10 mL.

*BMI* Body mass index, *CI* Confidence interval, *PON* Postoperative nausea, *POV* Postoperative vomiting, *PONV* Postoperative nausea and vomiting, *TIVA* Total intravenous anesthesia.

*P value < 0.05.

Considering the effect of different anesthesia methods on hemodynamics, the BP and HR after emergence were maintained to be lower in the TIVA group than in the inhalation anesthesia group. Furthermore, the hemodynamic response, such as the BP and HR, was more effectively controlled before and after emergence or extubation in the TIVA group than in the inhalation anesthesia group (BP; *P* < 0.001, HR; *P* = 0.024) (Table 5).

**Table 5:** Effect of anesthesia on hemodynamics.

	TIVA (n = 51)	Volatile anesthetics (n = 31)	OR (95% CI)	*P value*
Systolic blood pressure (mmHg)				
Pre	106.4 ± 9.6	107.7 ± 9.3	0.99 (0.94–1.03)	0.542
Average	102.3 ± 9.9	96.7 ± 11.1	1.06 (1.01–1.11)	0.023*
Peak (post)	125.9 ± 12.5	136.3 ± 12.0	0.93 (0.89–0.97)	0.002*
Diastolic blood pressure (mmHg)				
Pre	61.5 ± 7.1	65.0 ± 9.1	0.95 (0.89–1.00)	0.061
Average	53.5 ± 6.0	50.9 ± 6.2	1.07 (0.99–1.16)	0.076
Peak (post)	74.4 ± 9.5	81.9 ± 11.3	0.92 (0.89–0.98)	0.003*
Heart rate (min)				
Pre	76.5 ± 13.8	69.2 ± 10.1	1.05 (1.01–1.09)	0.016*
Average	62.1 ± 8.2	61.7 ± 8.7	1.00 (0.95–1.06)	0.867
Peak (post)	94.0 ± 14.6	101.2 ± 16.8	0.97 (0.941.00)	0.050
Ratio ((peak-average)/average)				
sBP	20.3 ± 9.8	33.9 ± 11.1	0.88 (0.83–0.93)	< 0.001*
dBP	40.1 ± 19.6	61.6 ± 19.9	0.95 (0.92–0.97)	< 0.001*
HR	39.6 ± 15.6	49.4 ± 19.9	0.97 (0.94–1.00)	0.024*

Mean ± standard deviation. *P value < 0.05.

*CI* Confidence interval, *OR* Odds ratio, *TIVA* Total intravenous anesthesia.

## Discussion

Several postoperative incidents have been reported in orthognathic surgery, and it is clinically important to manage the perioperative period and the surgical procedure. The management of PONV is critical during the postoperative period because PONV is the most common adverse event among anesthesia-related complications and can lead to serious complications^3,4^. Risk factors for the incidence of PONV are classified into three categories: patient-specific, anesthetic, and surgical factors. Patient-specific risk factors for PONV include female sex, young age, non-smoking status, obesity, and a history of PONV or motion sickness. Anesthetic or surgical risk factors for PONV include the type of surgery, duration of anesthesia and surgery, bleeding volume, use of volatile anesthetics, and postoperative use of opioids^3,6–8^. A high incidence of PONV has been noted among patients who have undergone orthognathic surgery.

Dobbeleir et al.^5^ reported that among 308 patients who underwent oral surgery, such as tooth extraction and temporomandibular joint surgery, including orthognathic surgery under general anesthesia, 46.1% experienced PON. However, 73.1% of patients who underwent orthognathic surgery experienced PON. Additionally, a high incidence of POV was observed among patients who underwent orthognathic surgery (39.4%), compared with the overall average of 21.1%. Phillips et al.^8^ reported a high incidence rate of PON (66.7%) and POV (27.0%) among patients who underwent orthognathic surgery. Laskin et al.^12^ also reported a high incidence of PON (42%) and POV (31%) among these patients. In the present study, the incidences of PON and POV were 45.1% and 19.5%, respectively, which were slightly lower than those reported previously; nonetheless, the results were similar.

A large proportion of young women undergo orthognathic surgery. Patient-specific and surgical factors are non-modifiable. However, anesthetic factors can be regulated. Therefore, identifying the anesthetic factors involved in PONV development is important. Inhalational anesthetics and opioid use are considered as anesthetic factors; nevertheless, the causal relationship regarding orthognathic surgery is unclear^7,8^. In the present study, the incidence of PON among patients who underwent orthognathic surgery was 31.4% in the TIVA group, accounting for approximately half of that in the inhalation anesthetic group (67.7%). The OR from the multivariate analysis was 5.3, suggesting that TIVA effectively prevented PON.

Female sex and a high infusion volume per body weight are risk factors for PON^8^. However, our study revealed that maintaining anesthesia with inhalation was the strongest risk factor for PON, and patient factors were not involved in multivariate analysis. In the stratified analysis of anesthesia method, a small amount of fluid infusion was strongly associated with PON when using TIVA. Preoperative fluid loading can reduce PONV based on the hypothesis that decreased blood flow to the intestinal tract during surgery increases serotonin release from the intestinal tract and subsequently induces vomiting^15^. It was suggested that sufficient fluid replacement during management with TIVA may further reduce the incidence of PON. However, Fearon et al.^16^ reported that excessive fluid load delayed intestinal edema and functional recovery in the Enhanced Recovery After Surgery Program. Hence, further studies are required to determine the optimal fluid volume. Using multivariate analysis, general anesthesia was the only risk factor for the development of PON, highlighting the usefulness of TIVA in orthognathic surgery.

In patients undergoing orthognathic surgery, race (non-Caucasian), morphine administration, and additional treatments, including removal of third molars, genioplasty, and mandibular bone harvest, have also been linked to the onset of POV^8^. It is generally known that intra- and postoperative opioid administration increases the risk of PONV^3,6^. In the present study, all participants were non-Caucasian, and no additional procedures, including tooth extraction, were performed. Additionally, we assessed the total intraoperative dose of opioids; cases of postoperative opioid administration were excluded. We found that the incidence of POV was high when the total dose of fentanyl was high; the cutoff value was 300 μg, suggesting that the amount of fentanyl used may influence the risk of POV. There is conflicting evidence on the relationship between BMI and PONV. One study found that PONV decreases in patients with high BMI^8^, while another study reported a weak association between BMI and PONV^2^. In our study, a high BMI increased the risk of POV in the volatile anesthesia group, as observed in the stratified analysis of anesthesia method, possibly due to accumulation in the adipose tissue. Our findings indicated that using TIVA may reduce the incidence of POV in cases of high BMI. Furthermore, in the present study, the statistical power of PON was adequate at approximately 90%. However, the power of POV was very low due to the generally low incidence of POV, making it challenging to obtain a sufficient sample size. Therefore, the focus here was on identifying and discussing the actual clinical situation regarding POV rather than statistical estimation. In future studies, we aim to expand the discussion by incorporating a study design with a larger sample size.

It is reported that the incidence of PONV does not differ between a single-jaw osteotomy and two-jaw surgery in patients undergoing orthognathic surgery^5,8^. However, since the incidence of PONV increases with longer surgery duration, verifying the usefulness of TIVA for two-jaw surgery is important. We included only the sagittal split ramus osteotomy procedure in the present study due to the small sample size. Thus, further research will be necessary, including two-jaw surgery and other surgical techniques. Additionally, we could not investigate the involvement of a history of PONV or motion sickness, which are important risk factors for PONV; hence, further investigation is necessary in the future. Although not feasible in the present study, we aim to deepen the discussion in future research by considering a study design with a larger sample size, potentially including a multicenter study.

PONV is mainly caused by stimulation of the vomiting center via the chemoreceptor trigger zone (CTZ) in the area postrema of the medulla oblongata. The CTZ receptors include serotonin, histamine, dopamine, and substance P receptors^17,18^. In addition to CTZ, there are three inputs to the vomiting center: higher brain centers, the vestibular organ, and the vagal afferent pathway. Therefore, it is possible to prevent PONV by using antagonists of each receptor, such as 5-HT3 receptor antagonists, antihistamines, corticosteroids, dopamine antagonists, NK-1 receptor antagonists, and anticholinergics^2^. In our department, dexamethasone is administered preoperatively and the day after surgery to prevent postoperative swelling and PONV. Droperidol has been reported to reduce POV in orthognathic surgery^8^. Moreover, prevention of PONV by perioperative multimodal medication is an area of future research in all cases.

EA is less likely to occur after TIVA compared with after volatile anesthesia^10,11^. Increased BP and HR due to EA and tracheal extubation are common^19^, which lead to complications, such as postoperative bleeding and myocardial ischemia. Although the use of beta-blockers has been shown to suppress cardiovascular changes, such as hypertension and tachycardia, during extubation^20^, there are no comparative studies of TIVA and volatile anesthesia. Our study demonstrated that changes in hemodynamics, such as the BP and HR, during extubation and emergence from anesthesia were less pronounced in the TIVA group than in the volatile anesthesia group. Moreover, in the present study, preoperative HR was higher in the TIVA group than in the volatile anesthesia group. HR has been reported to be higher in women than in men and in younger patients than in older patients^21–23^. The patient population in our study tended to include more female and younger patients in the TIVA group, which may have influenced the preoperative HR in the present study. In any case, the TIVA group exhibited stabilized HR variability after extubation compared with the inhalational anesthesia group. Although there were no complications, such as bleeding after extubation, in either group, TIVA was considered useful in terms of maintaining hemodynamic stability.

Due to the retrospective nature of the present study and inherent biases in medical record reviews, future research should focus on adjusting for background factors through prospective studies and randomized controlled trials to explore underlying mechanisms. Additionally, conducting multicenter studies with larger sample sizes would be beneficial.

## Conclusions

General anesthesia was the strongest risk factor for PONV in patients undergoing orthognathic surgery. Our results indicated that anesthesia management with TIVA in patients undergoing orthognathic surgery could reduce PONV and the hemodynamic changes during extubation and emergence from anesthesia. Moreover, given the perioperative complications of orthognathic surgery, using TIVA for anesthetic management showed promise. Thorough consideration of the choice of anesthesia may include providing information to patients prior to surgery, including perioperative complications. It is hoped that a prospective randomized controlled trial will be conducted in the future to enable a more detailed analysis, including elucidation of the underlying mechanisms.

### Supplementary Information

Supplementary Information.

## Data Availability

The datasets used and/or analyzed during the current study are available from the corresponding author on reasonable request.

## Abbreviations

BP: Blood pressure
CI: Confidence interval
CTZ: Chemoreceptor trigger zone
EA: Emergence agitation
HR: Heart rate
OR: Odds ratio
PON: Postoperative nausea
PONV: Postoperative nausea and vomiting
TIVA: Total intravenous anesthesia
