# Supplementary material for: Impact of general anesthesia on postoperative complications in orthognathic surgery: a retrospective comparison of total intravenous anesthesia versus volatile anesthesia
Source: Sci Rep. 2024 Jul 12;14:16075. doi: 10.1038/s41598-024-66926-w (PMC11239665; doi:10.1038/s41598-024-66926-w)
Supplement: Supplementary file 1 — Supplementary Information. [file 41598_2024_66926_MOESM1_ESM.docx]

***Supplementary Table S1. Volatile anesthetics concentration used for maintenance***

| Volatile anesthetics (number) |  | Range (vol%) |  | Average concentration (vo1%) |  | S.D. |
| --- | --- | --- | --- | --- | --- | --- |
| Sevoflurane　(29) |  | 1.0-2.0 |  | 1.41 |  | 0.15 |
| Desflurane 　(2) |  | 5.0-6.0 |  | 5.25 |  | 0.35 |

S.D., standard deviation
